# Multi-level Cells and Quantized Conductance Characteristics of Al_2_O_3_-Based RRAM Device for Neuromorphic System

**DOI:** 10.1186/s11671-022-03722-3

**Published:** 2022-09-03

**Authors:** Yunseok Lee, Jongmin Park, Daewon Chung, Kisong Lee, Sungjun Kim

**Affiliations:** 1 Division of Electronics and Electrical Engineering, Dongguk University, Seoul, 04620 Republic of Korea; 2 Department of Information and Communication Engineering, Dongguk University, Seoul, 04620 Republic of Korea

**Keywords:** Neuromorphic system, Memristor, Al_2_O_3_, Quantized conductance, MLC

## Abstract

Recently, various resistance-based memory devices are being studied to replace charge-based memory devices to satisfy high-performance memory requirements. Resistance random access memory (RRAM) shows superior performances such as fast switching speed, structural scalability, and long retention. This work presented the different filament control by the DC voltages and verified its characteristics as a synaptic device by pulse measurement. Firstly, two current–voltage (*I*–*V*) curves are characterized by controlling a range of DC voltages. The retention and endurance for each different *I*–*V* curve were measured to prove the reliability of the RRAM device. The detailed voltage manipulation confirmed the characteristics of multi-level cell (MLC) and conductance quantization. Lastly, synaptic functions such as potentiation and depression, paired-pulse depression, excitatory post-synaptic current, and spike-timing-dependent plasticity were verified. Collectively, we concluded that Pt/Al_2_O_3_/TaN is appropriate for the neuromorphic device.

## Introduction

In an environment where data demand is rapidly increasing, a breakthrough is needed in computing performance limitations due to serial processing of CPU and memory [1]. It is necessary to change the computing structure and improve the materials of the memory device to solve the memory wall. Neuromorphic computing architecture is emerging as a structural solution to the bottleneck. The neuromorphic computing system mimics the neuron and synapses of the human brain [2–4]. This system is suitable for the process of complex and unstructured information. First of all, to implement neuromorphic computing, it is necessary to understand how the human brain processes information. The human brain includes numerous synapses and neurons, and learning and memory of information proceed through parallel chemical interactions. Information processing and memory capabilities vary depending on various factors such as the size, holding time, and a repetition time of external signals and stimuli [5–7].

Among various memories, the RRAM exhibits a fast switching speed and a low operating voltage [8–15]. In addition, RRAM could be implemented in a simple structure such as a metal-oxide-metal (MIM) with various structural expandability [16–21] such as the connection of transistor with each memory cell, an array structure, and a 3D vertical structure.

The switching of RRAM occurs by the formation and rupture of filament in an insulator between the metals [22–26]. The resistance of RRAM is varied through a conductive filament composed of oxygen vacancy in the insulator existing between the top electrode (TE) and bottom electrode (BE) and has two basic switching states (high and low) to process the data storage process [20, 27–30]. In the case of the high-resistance state (HRS), a low current flows in HRS, and in the case of the low-resistance state (LRS), it means a state has low resistance and good conductivity. Accordingly, the on/off state could be monitored through the read voltage. The repetition of set and reset processes cause the device to move back and forth between the HRS and LRS, which can be described as a memory that stores 0 and 1 from a digital perspective.

In this paper, the gradual resistive switching is conducted on Pt/Al_2_O_3_/TaN device, including Al_2_O_3_ high-k dielectric [31–35], which was deposited by atomic layer deposition (ALD) equipment. The characteristics using basic DC current sweep and on/off endurance characteristics were measured, and the suitability of neuromorphic devices was also measured through synaptic measurement, including potentiation, depression, PPD, EPSC, and STDP.

## Experiments

Pt/Al_2_O_3_/TaN device was fabricated as follows. Firstly, TaN as BE was deposited by the sputtering system on SiO_2_/Si wafer. A 5-nm-thick Al_2_O_3_ film was deposited by the ALD process. In the ALD process, TMA precursors and O_3_ were used at stage temperature 450 °C. Then a 100-nm-thick Pt as TE was deposited by evaporator in which the top pattern was formed in a circular pattern by using a shadow mask with a diameter of 100 µm. For the measurement environment, all measurements were performed at room temperature and ambient atomic pressure. Electrical data were measured using the Keithley 4200-SCS semiconductor parameter ultrafast module and in pulse mode using a 4225-PMU ultrafast module.

## Results and Discussion

**Fig. 1:**
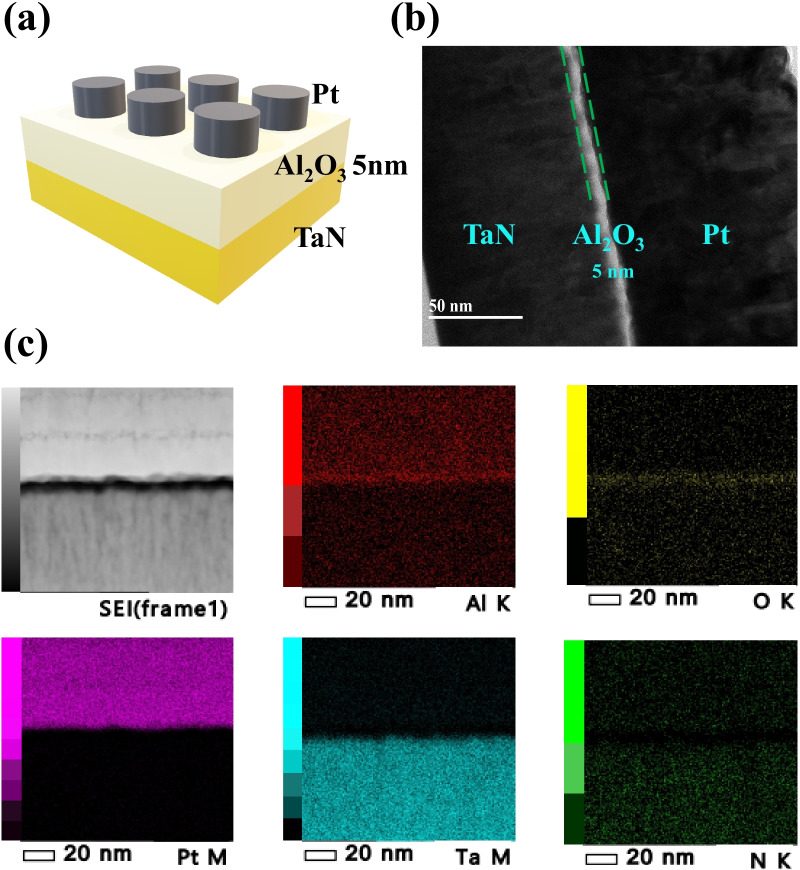
**a** Schematics image of Pt/Al_2_O_3_/TaN device, **b** cross-sectional TEM image, **c** EDS mapping images of Pt, Al, O, Ta and N elements collected from the area indicated in the TEM image of the Pt/Al_2_O_3_/TaN device

Figure 1a shows the schematic illustration of the fabricated Pt/Al_2_O_3_/TaN device. In Fig. 1b, the cross section of the Pt/Al_2_O_3_/TaN RRAM device is inspected by a transmission electron microscope (TEM). The thickness of the Al_2_O_3_ insulator layer deposited by the ALD system is about 5 nm. In Fig. 1c, energy dispersion X-ray spectroscopy (EDS) mapping of each element was performed to investigate possible chemical interactions. EDS mapping shows the spatial distribution of elements in Pt/Al_2_O_3_/TaN. EDS maps of Pt, Al, O, Ta, and N elements were collected in the area shown in the electronic image. A region where O and Ta overlap is observed, indicating The TaON interface layer between the Al_2_O_3_ insulator and TaN BE is formed by a chemical redox reaction between the TaN BE and the lower Al_2_O_3_ layer due to the strong oxygen binding of TaN [36–38]. Because of the formation of the TaON interface layer by extracting oxygen from the Al_2_O_3_ layer by TaN, better switching characteristics could be exhibited according to the formation of the oxygen vacancy near the TaON/Al_2_O_3_ interface [38].

**Fig. 2:**
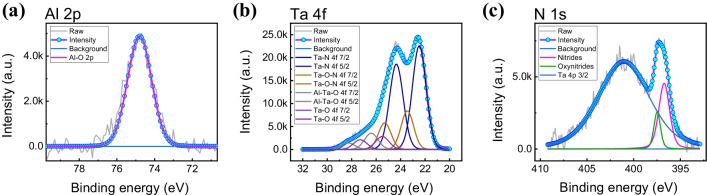
XPS spectra of **a** Al 2*p*, **b** Ta 4*f* and **c** N 1*s* of the device

In order to confirm the TaON layer, the X-ray photoelectron spectroscopy (XPS) spectra fittings were conducted. Figure 2a shows the Al 2*p* XPS spectra in which peak intensity is located at 75 eV for Al–O bonding [39]. Figure 2b and c shows Ta 4*f* and N 1*s* XPS peak for the TaON layer. In Fig. 2b, small peaks exist at higher binding energy than general Ta 4*f* XPS peaks. This indicates that the binding Ta–O or Ta–Al energy also affected the Ta 4*f* XPS peaks with binding Ta–N energy [40, 41]. From Fig. 2c, through combination with oxygen, N 1*s* XPS peak shows more biased to higher binding energy than the normal N 1*s* peak [42]. As a result, a thin layer of TaON exists between the Al_2_O_3_ insulator and the TaN BE.

**Fig. 3:**
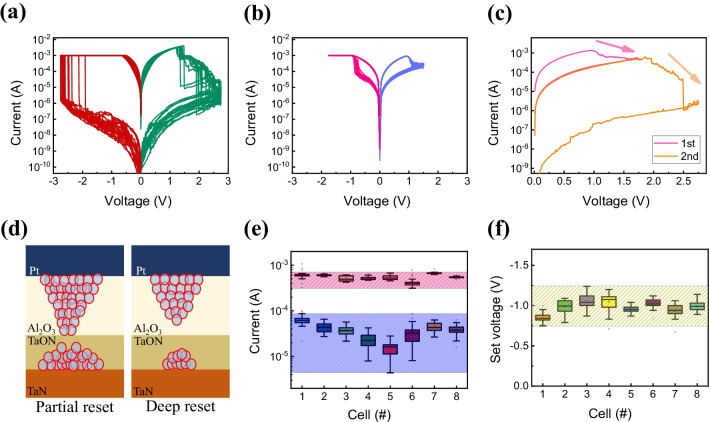
Bipolar resistive switching of Pt/Al_2_O_3_/TaN device: **a** deep reset, **b** partial reset, **c** process of inducing from partial reset to deep reset, and **d** deep and partial reset mechanism schematic diagram. Uniformity of **e** HRS and LRS, **f** set voltage

Next, we investigate two types of bipolar resistive switching by DC sweep. All of the above *I*–*V* characteristics were measured at a step voltage of 0.01 V. Representative feature of this device shows forming-free characteristics in Fig. 3a [43]. The set process has similar *I*–*V* curves as the forming process, and the set process occurs at − 2 V or higher, and the reset process is induced by applying a 2.75 V. This is referred to as a deep reset curve. At this time, the on/off ratio is about 45,000 based on the read voltage of 0.5 V, which is a characteristic due to a large band gap of Al_2_O_3_. Set shows abrupt behavior, and in the reset process, it shows a curve that returns to the HRS state with a stepwise drop from 1 V or higher to 2.7 V. In the case of Fig. 3b, unlike Fig. 3a, it can be implemented by adjusting reset voltage less than 2.75 V. This is referred to as a partial reset curve, and the on/off ratio at this time is about 13 at the read voltage of 0.5 V. Compared to the *I*–*V* curves with fully reset, the *I*–*V* curves with partial reset process shows more gradual characteristics in the set and reset processes. Both *I*–*V* characteristics have self-compliance characteristics [44]. The method of connecting the two differences in Fig. 3a and b can be confirmed by a continuous DC sweep in Fig. 3c. The deep reset occurs when the larger voltage is applied, indicating that the strength of the reset can be controlled by the voltage adjustment. The current flows in the HRS induced by the partial reset and an additional reset occur, which lowers the current level due to additional filament decomposition. Figure 3d exhibits a possible switching mechanism of partial reset (left) and deep reset (right) curves. As confirmed in Fig. 2, Al–O bonding has higher binding energy than that Ta–O bonding. This suggests that switching depends on the TaON layer when the small electric field is applied and on the Al_2_O_3_ layer when it is a large electric field. Thus, oxygen ions formed between TaN and TaON affect the conduction mechanism of the device and are estimated to result in MLC characteristics [3, 45, 46]. Gradual partial reset with MLC occurs in the TaON layer within the − 2.2 V region. However, the more electric field induces the filament decomposition inside the Al_2_O_3_ and causes the abrupt current decrease during the reset process. In Fig. 3e, HRS and LRS were confirmed in the read operation of 0.5 V to demonstrate state uniformity. Since the filament decomposition depends on the magnitude of reset voltage, HRS varies more severely than LRS. Also, more decomposition demands more set voltage to re-form the filaments. Variation of set voltage is shown in Fig. 3f and it varied from − 1.25 to − 0.75 V in accordance with the previous reset cycle process.

**Fig. 4:**
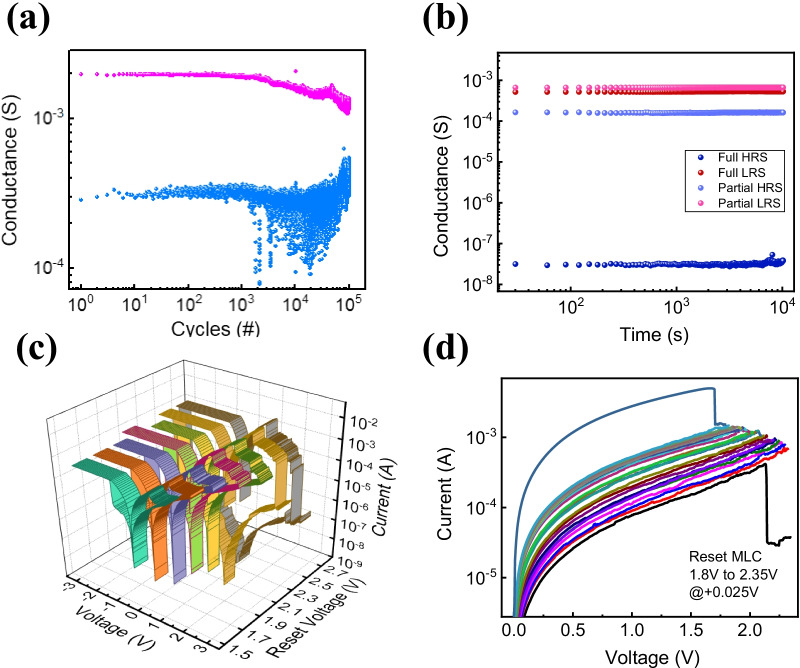
**a** Endurance characteristic of the device for 10^5^ cycles and **b** each deep and partial HRS and LRS retention characteristics for 10^4^ s. MLC characteristics of **c** different reset voltages, **d** consecutive incremental voltage repetition

In Fig. 4a, the endurance characteristics were also measured for partial *I*–*V* conditions using pulse for 10^5^ cycles. It shows that HRS and LRS can be switched even at 10^5^ or more times. In Fig. 4b, it is the result of performing the retention test for each *I*–*V* characteristic including partial and deep resets. HRS and LRS were measured at the read voltage of 0.15 V, and both states were maintained for 10^4^ s. These results show the Pt/Al_2_O_3_/TaN device has good non-volatile memory properties. Multi-level cell characteristics are very beneficial for practical applications such as high-density memory and neuromorphic device [43, 47, 48]. Figure 4c shows a reset process by increasing the reset voltage by 0.2 V for each cycle. Through this process, as the reset voltage increases, multiple HRS is achieved. In Fig. 4d, based on the reset voltage at the boundary between the partial reset and deep reset, the reset process was repeatedly measured while increasing 0.025 V from 1.8 to 2.35 V. It could be verified that the current level gradually decreases, and this could prove the existence of various multi-level states.

**Fig. 5:**
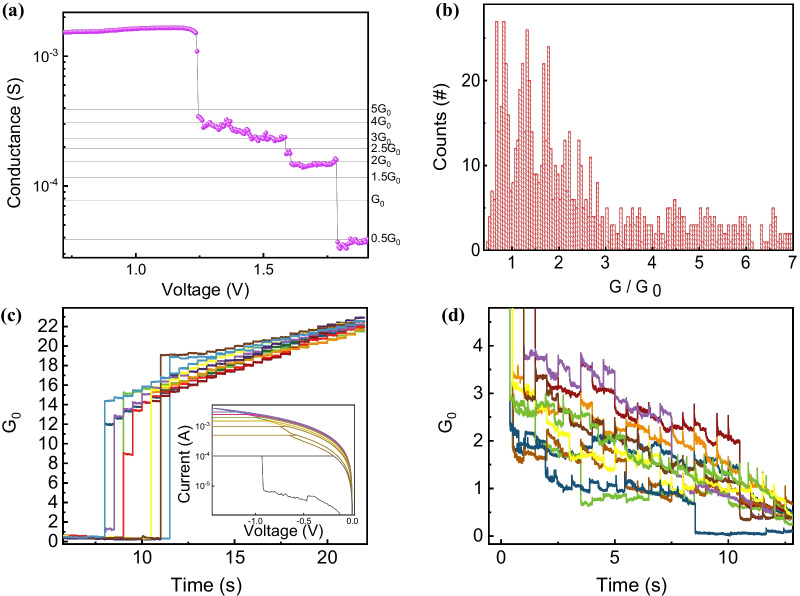
**a** Quantized conductance characteristic using DC measurement on reset part, and **b** histogram of quantized conductance levels. Quantized conductance characteristic using pulse measurement **c** in set region, **d** in reset region

The property of conductance quantization [49–52] was confirmed. This is thought to be due to the quantization effect of conductive filament during the reset process. When the conductive filament is well controlled, it is possible to implement more state and higher density memory through this phenomenon. As shown in Fig. 5a, this phenomenon can be observed when the conductive filament is modified in atomic units. The step voltage of 0.002 V and delay time of 0.3 s every step is used to observe quantization in multiple cycles, and only elemental disruption of the filament was measured during the reset process. The conductance quantum, represented by the symbol *G*_0_, is the quantized unit of electrical conductance. It is defined by the elementary charge *e* and Planck constant *h* as *G*_0_ = 2*e*^2^/*h* = 7.74809 × 10^–5^ S. The device takes an integer multiple of *G*_0_ or an intermediate value between integers. In the end, LRS is changed to HRS. The statistical analysis is essential through multiple cycles [53–57]. Figure 5b shows the histogram plotting, and it can be seen that even in various conductance steps, there is a high tendency near a multiple of *G*_0_ or a half multiple [58–61]. It is noted the values between 0.5*G*_0_ and 3*G*_0_ are distinctly distinguishable. It may be necessary to make the conducting filament smaller by means of a method such as making the device smaller in order to distinguish the quantized values. Pulse measurements were performed in Fig. 5c and d to describe the quantized conductance [62–64]. Conductance calculated with the voltage of 0.5 V was induced by adding write pulses at 0.5 s intervals. In Fig. 5c, an incremental write pulse increased by − 25 mV from − 0.7 to − 1.775 V was used and the abrupt set operation occurred at a voltage of − 1 V or higher. The conductance in HRS increases more than 10*G*_0_ at a time due to the abrupt characteristic in the set region. This characteristic was also confirmed in the *I*–*V* curve in the inset image of Fig. 5c, which MLC implemented by limiting compliance current. In contrast, conductance quantization with the erase pulses composed by 25 mV from 1.5 to 2.175 V were ranged of about *G*_0_. From those two different conductance ranges show that it is more ease to implement MLC during reset process due to the clear state distinction.

**Fig. 6:**
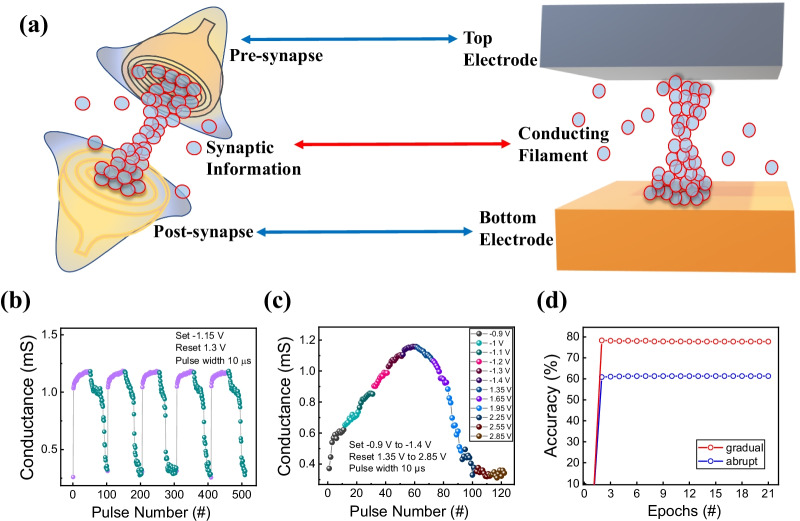
**a** Schematic illustration of similarity between human synaptic neural structure and RRAM device structure. Potentiation and depression characteristics: **b** consecutive same voltage pulse, **c** single incremental voltage pulse. **d** MNIST pattern recognition simulation results of (**b**) and (**c**)

A neuromorphic computing system can be implemented using multi-level cells in Pt/Al_2_O_3_/TaN devices. As shown in Fig. 6a, the conductive filament connecting the TE and BE of RRAM can be expressed very similarly to the human’s biological system [43, 65–67]. In order to confirm the suitability of neuromorphic computing, pulse measurements were conducted. In Fig. 6b, conductance control is continuously performed through 5 cycles of potentiation and depression by applying the pulses. Potentiation and depression were set to − 1.15 V and 1.3 V, respectively, and both pulse widths were set to 10 sµ. From the *I*–*V* characteristic of the set process, relatively abruptness in the potentiation can be confirmed. It could be verified that the depression part has a more gradual characteristic. Moreover, we demonstrate more gradual and symmetric resistance-change characteristics by controlling the voltage amplitude of pulses in Fig. 6c [68, 69]. Each 6 potentiation and depression segments are used to increase and decrease the conductance. The voltage varied from − 0.9 to − 1.4 V for potentiation and from 1.35 to 2.85 V for depression. Figure 6d shows MNIST pattern recognition simulation results by using the conductance results of Fig. 6b and c [70, 71]. The result of using Fig. 6c shows higher accuracy for each epoch. In other words, pulse improvement measurement provides a better learning process.

**Fig. 7:**
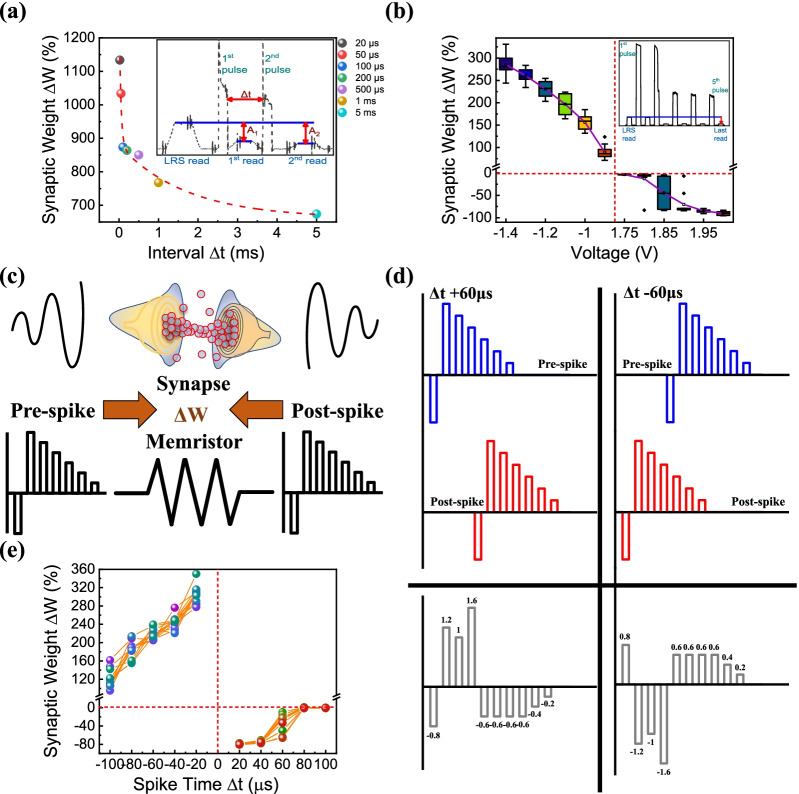
**a** PPD measurement, **b** EPSC data according to amplitude. STDP characteristics: **c** Schematic for measurement imitation between synaptic neural structure and RRAM, **d** pulse authorization for STDP measurement at Δ*t* = 60 μs and **e** result of STDP measurement

Synaptic functions, such as PPD, EPSC, and STDP measurements, were performed to determine suitability for the neuromorphic application [72–74]. Figure 7a shows the device's PPD measurement data, the ratio change between two pulses was confirmed when the seven different intervals were used. Synaptic weight changed with the time interval ranging from 20 μs to 5 ms between two consecutive depression pulses. The amount of synaptic weight change was expressed as Δ*W* = (*A*_2_ − *A*_1_)/*A*_1_ × 100 (%). As a result, the current responded by the second pulse decreases as the interval increases, indicating that the device is suitable for implementing STP. Figure 7b illustrates conductance changes before and after giving five identical write pulses and summarizes them with pulse amplitudes. As the voltage amplitude increases, both potentiation and depression have a larger synaptic weight change. Continuous stimulation raises EPSC; the degree of weight strengthening can be adjusted according to the amplitude. The strength of connections between neurons in biological synapses can be controlled by STDP. Therefore, if we can elucidate the detailed mechanisms of biological synaptic action and imitate the action behavior, it will be possible to mimic the energy-efficient processing of the human brain. Figure 7c explains the configuration of the STDP protocol. When the pre-spike signal and post-spike signal, which vary with the interval, were applied to the biological synapses, the weight was changed and implemented according to the learning behavior. This process was mimicked on the memristor in the same way. The pulse protocol in Fig. 7d was used for the measurements. The same pre and post-signal were composed, but the different shape of pulses was finally configured and applied according to the interval. Since the final pulse configuration was different, the synaptic weights over time had different weight changes, as shown in Fig. 7e [2, 75, 76]. In general, the shorter the absolute time of the spike time difference, the greater the change in conductance change like a biological synapse.

## Conclusions

As a result, the MLC characteristics and quantized conductance were confirmed through the Al_2_O_3_-based RRAM device deposited with ALD, and excellent biological characteristics were investigated through pulse measurement. DC *I*–*V* bipolar switching characteristics were verified through DC measurement, and it was verified that switching characteristics of two different characteristics could be easily controlled only by adjusting a voltage. Multi-levels in various cases were confirmed by varying the amount of voltage that adjusts different characteristics, and the conductance quantization phenomenon was also confirmed within the reset section and pulse measurements. This MLC phenomenon was connected with pulse measurement to measure potentiation and depression, and it was possible to maximize MLC characteristics through voltage control of each segment. Including PPD and EPSC, through the measurement of STDP, the change in the conductance weight of the device was confirmed by imitating the synapse. In conclusion, the MLC characteristics of the device and the suitability of neuromorphic computing were successfully completed.

## Data Availability

All data generated or analysed during this study are included in this article.

## References

[1] Wang R, Yang J-Q, Mao J-Y (2020). Recent advances of volatile memristors: devices, mechanisms, and applications. Adv Intell Syst.

[2] Kim S, Kim H, Hwang S (2017). Analog synaptic behavior of a silicon nitride memristor. ACS Appl Mater Interfaces.

[3] Ismail M, Abbas H, Sokolov A (2021). Emulating synaptic plasticity and resistive switching characteristics through amorphous Ta_2_O_5_ embedded layer for neuromorphic computing. Ceram Int.

[4] Li Q, Tao Q, Chen Y (2021). Low voltage and robust InSe memristor using van der Waals electrodes integration. Int J Extreme Manuf.

[5] Ryu H, Kim S (2020). Self-rectifying resistive switching and short-term memory characteristics in Pt/HFO_2_/TaO_x_/TiN artificial synaptic device. Nanomaterials.

[6] Park M, Kang M, Kim S (2021). Pulse frequency dependent synaptic characteristics in Ta/SiN/Si memristor device for neuromorphic system. J Alloys Compd.

[7] Ryu JH, Mahata C, Kim S (2021). Long-term and short-term plasticity of Ta_2_O_5_/HfO_2_ memristor for hardware neuromorphic application. J Alloys Compd.

[8] Kim D, Kim S, Kim S (2021). Logic-in-memory application of CMOS compatible silicon nitride memristor. Chaos Solitons Fract.

[9] Lin KL, Hou TH, Shieh J (2011). Electrode dependence of filament formation in HfO_2_ resistive-switching memory. J Appl Phys.

[10] Rodriguez-Fernandez A, Aldana S, Campabadal F (2017). Resistive switching with self-rectifying tunability and influence of the oxide layer thickness in Ni/HfO_2_/n^+^-Si RRAM devices. IEEE Trans Electron Devices.

[11] Hu G, An H, Xi J (2021). A ZnO micro/nanowire-based photonic synapse with piezo-phototronic modulation. Nano Energy.

[12] Khan SA, Lee GH, Mahata C (2021). Bipolar and complementary resistive switching characteristics and neuromorphic system simulation in a Pt/ZnO/TiN synaptic device. Nanomaterials.

[13] Sun J, Tan JB, Chen T (2020). Investigation of electrical noise signal triggered resistive switching and its implications. IEEE Trans Electron Devices.

[14] Shin J, Kang M, Kim S (2021). Gradual conductance modulation of Ti/WO_x_/Pt memristor with self-rectification for a neuromorphic system. Appl Phys Lett.

[15] Chen ZX, Fang Z, Wang Y (2014). Impact of Ni concentration on the performance of Ni silicide/HfO_2_/TiN resistive RAM (RRAM) cells. J Electron Mater.

[16] Luo Q, Xu X, Gong T et al (2018) 8-Layers 3D vertical RRAM with excellent scalability towards storage class memory applications. In: Technical digest—international electron devices meeting, IEDM

[17] Yu S, Chen HY, Gao B (2013). HfO_x_-based vertical resistive switching random access memory suitable for bit-cost-effective three-dimensional cross-point architecture. ACS Nano.

[18] Al-Haddad A, Wang C, Qi H (2016). Highly-ordered 3D vertical resistive switching memory arrays with ultralow power consumption and ultrahigh density. ACS Appl Mater Interfaces.

[19] Yu M, Cai Y, Wang Z (2016). Novel vertical 3D structure of TaO_x_-based RRAM with self-localized switching region by sidewall electrode oxidation. Sci Rep.

[20] Jeong DS, Thomas R, Katiyar RS (2012). Emerging memories: resistive switching mechanisms and current status. Rep Prog Phys.

[21] Lanza M, Wong HSP, Pop E (2019). Recommended methods to study resistive switching devices. Adv Electron Mater.

[22] Waser R, Aono M (2007). Nanoionics-based resistive switching memories. Nat Mater.

[23] Sawa A (2008). Resistive switching in transition metal oxides. Mater Today.

[24] Yu S (2014) Overview of resistive switching memory (RRAM) switching mechanism and device modeling. In: Proceedings—IEEE international symposium on circuits and systems

[25] Li YT, Long SB, Liu Q (2011). An overview of resistive random access memory devices. Chin Sci Bull.

[26] Choi J, Kim S (2020). Improved stability and controllability in ZrN-based resistive memory device by inserting TiO_2_ layer. Micromachines.

[27] Kim S, Chen J, Chen YC (2019). Neuronal dynamics in HfO_x_/AlO_y_-based homeothermic synaptic memristors with low-power and homogeneous resistive switching. Nanoscale.

[28] Zhang K, Sun K, Wang F (2015). Ultra-low power Ni/HfO_2_/TiO_x_/TiN resistive random access memory with sub-30-nA reset current. IEEE Electron Device Lett.

[29] Sun QQ, Gu JJ, Chen L (2011). Controllable filament with electric field engineering for resistive switching uniformity. IEEE Electron Device Lett.

[30] Kwon O, Kim Y, Kang M, Kim S (2021). Comparison of synaptic properties considering dopant concentration and device operation polarity in Cu/SiN/SiO_2_/p-Si devices for neuromorphic system. Appl Surf Sci.

[31] Vishwanath SK, Woo H, Jeon S (2018). Enhancement of resistive switching properties in Al_2_O_3_ bilayer-based atomic switches: Multilevel resistive switching. Nanotechnology.

[32] Chen L, Gou HY, Sun QQ (2011). Enhancement of resistive switching characteristics in Al_2_O_3_-based RRAM with embedded ruthenium nanocrystals. IEEE Electron Device Lett.

[33] Chen L, Xu Y, Sun QQ (2010). Highly uniform bipolar resistive switching with Al_2_O_3_ buffer layer in robust NbAlO-based RRAM. IEEE Electron Device Lett.

[34] Kim S, Park BG (2016). Nonlinear and multilevel resistive switching memory in Ni/Si_3_N_4_/Al_2_O_3_/TiN structures. Appl Phys Lett.

[35] Chen C, Pan F, Wang ZS (2012). Bipolar resistive switching with self-rectifying effects in Al/ZnO/Si structure. J Appl Phys.

[36] Wang Z, Yaegashi O, Sakaue H (2003). Suppression of native oxide growth in sputtered TaN films and its application to Cu electroless plating. J Appl Phys.

[37] Zhou Q, Zhai J (2014). Study of the bipolar resistive-switching behaviors in Pt/GdO_x_/TaN_x_ structure for RRAM application. Physica Status Solidi A Appl Mater Sci.

[38] Zhou P, Yin M, Wan HJ (2009). Role of TaON interface for Cu_x_O resistive switching memory based on a combined model. Appl Phys Lett.

[39] Fang R-C, Sun Q-Q, Zhou P (2013). High-performance bilayer flexible resistive random access memory based on low-temperature thermal atomic layer deposition. Nanoscale Res Lett.

[40] Zhou J, dan Nie D, Jin XB, Xiao W (2020). Controllable nitridation of Ta_2_O_5_ in molten salts for enhanced photocatalysis. Int J Miner Metall Mater.

[41] Misha SH, Tamanna N, Woo J (2015). Effect of nitrogen doping on variability of TaO_x_-RRAM for low-power 3-bit MLC applications. ECS Solid State Lett.

[42] Cristea D, Cunha L, Gabor C (2019). Tantalum oxynitride thin films: assessment of the photocatalytic efficiency and antimicrobial capacity. Nanomaterials.

[43] Ismail M, Mahata C, Kim S (2022). Forming-free Pt/Al_2_O_3_/HfO_2_/HfAlO_x_/TiN memristor with controllable multilevel resistive switching and neuromorphic characteristics for artificial synapse. J Alloys Compd.

[44] Ryu H, Kim S (2021). Gradually modified conductance in the self-compliance region of an atomic-layer-deposited Pt/TiO_2_/HfAlO_x_/TiN rram device. Metals.

[45] Chen MC, Chang TC, Chiu YC (2013). The resistive switching characteristics in TaON films for nonvolatile memory applications. Thin Solid Films.

[46] Ismail M, Abbas H, Mahata C (2022). Optimizing the thickness of Ta_2_O_5_ interfacial barrier layer to limit the oxidization of Ta ohmic interface and ZrO_2_ switching layer for multilevel data storage. J Mater Sci Technol.

[47] Lin J, Wang S, Liu H (2021). Multi-level switching of al-doped HfO_2_ RRAM with a single voltage amplitude set pulse. Electronics (Switzerland).

[48] Wu J, Ye C, Zhang J (2016). Multilevel characteristics for bipolar resistive random access memory based on hafnium doped SiO_2_ switching layer. Mater Sci Semicond Process.

[49] Li Y, Long S, Liu Y (2015). Conductance quantization in resistive random access memory. Nanoscale Res Lett.

[50] Celano U, Goux L, Belmonte A (2015). Understanding the dual nature of the filament dissolution in conductive bridging devices. J Phys Chem Lett.

[51] Sharath SU, Vogel S, Molina-Luna L (2017). Control of switching modes and conductance quantization in oxygen engineered HfO_x_ based memristive devices. Adv Funct Mater.

[52] Park J, Lee S, Lee K, Kim S (2021). Conductance quantization behavior in pt/sin/tan rram device for multilevel cell. Metals.

[53] Sun Y, Wen D (2018). Conductance quantization in nonvolatile resistive switching memory based on the polymer composite of zinc oxide nanoparticles. J Phys Chem C.

[54] Gao S, Zeng F, Chen C (2013). Conductance quantization in a Ag filament-based polymer resistive memory. Nanotechnology.

[55] Zhu X, Su W, Liu Y (2012). Observation of conductance quantization in oxide-based resistive switching memory. Adv Mater.

[56] Mehonic A, Vrajitoarea A, Cueff S (2013). Quantum conductance in silicon oxide resistive memory devices. Sci Rep.

[57] Gao S, Chen C, Zhai Z (2014). Resistive switching and conductance quantization in Ag/SiO_2_/indium tin oxide resistive memories. Appl Phys Lett.

[58] Long S, Lian X, Cagli C (2013). Quantum-size effects in hafnium-oxide resistive switching. Appl Phys Lett.

[59] Tsuruoka T, Hasegawa T, Terabe K, Aono M (2012). Conductance quantization and synaptic behavior in a Ta_2_O_5_-based atomic switch. Nanotechnology.

[60] Chen C, Gao S, Zeng F (2013). Conductance quantization in oxygen-anion-migration-based resistive switching memory devices. Appl Phys Lett.

[61] Lv H, Xu X, Sun P (2015). Atomic view of filament growth in electrochemical memristive elements. Sci Rep.

[62] Milano G, Aono M, Boarino L (2022). Quantum conductance in memristive devices: fundamentals, developments, and applications. Adv Mater.

[63] Zhao X, Xu J, Xie D (2021). Natural acidic polysaccharide-based memristors for transient electronics: highly controllable quantized conductance for integrated memory and nonvolatile logic applications. Adv Mater.

[64] Banerjee W, Hwang H (2019). Quantized conduction device with 6-bit storage based on electrically controllable break junctions. Adv Electron Mater.

[65] Strukov DB, Kohlstedt H (2012). Resistive switching phenomena in thin films: Materials, devices, and applications. MRS Bull.

[66] Zhang SR, Zhou L, Mao JY (2019). Artificial synapse emulated by charge trapping-based resistive switching device. Adv Mater Technol.

[67] Rahmani MK, Ismail M, Mahata C, Kim S (2020). Effect of interlayer on resistive switching properties of SnO_2_-based memristor for synaptic application. Results Phys.

[68] Park J, Ryu H, Kim S (2021). Nonideal resistive and synaptic characteristics in Ag/ZnO/TiN device for neuromorphic system. Sci Rep.

[69] Cho H, Kim S (2020). Enhancing short-term plasticity by inserting a thin TiO_2_ layer in WO_x_-based resistive switching memory. Coatings.

[70] Ismail M, Mahata C, Kwon O, Kim S (2022). Neuromorphic synapses with high switching uniformity and multilevel memory storage enabled through a Hf-Al-O alloy for artificial intelligence. ACS Appl Electron Mater.

[71] Lee Y, Mahata C, Kang M, Kim S (2021). Short-term and long-term synaptic plasticity in Ag/HfO_2_/SiO_2_/Si stack by controlling conducting filament strength. Appl Surf Sci.

[72] Feng G, Jiang J, Zhao Y (2020). A sub-10 nm vertical organic/inorganic hybrid transistor for pain-perceptual and sensitization-regulated nociceptor emulation. Adv Mater.

[73] Li Y, Yin K, Diao Y (2022). A biopolymer-gated ionotronic junctionless oxide transistor array for spatiotemporal pain-perception emulation in nociceptor network. Nanoscale.

[74] Jiang J, Hu W, Xie D (2019). 2D electric-double-layer phototransistor for photoelectronic and spatiotemporal hybrid neuromorphic integration. Nanoscale.

[75] Shen Z, Zhao C, Qi Y (2020). Advances of RRAM devices: Resistive switching mechanisms, materials and bionic synaptic application. Nanomaterials.

[76] Lu K, Li Y, He WF (2018). Diverse spike-timing-dependent plasticity based on multilevel HfOx memristor for neuromorphic computing. Appl Phys A Mater Sci Process.

